# Detection of Hepatitis B Virus M204I Mutation by Quantum Dot-Labeled DNA Probe

**DOI:** 10.3390/s17050961

**Published:** 2017-04-26

**Authors:** Cheng Zhang, Yiping Chen, Xinmiao Liang, Guanhua Zhang, Hong Ma, Leng Nie, Yu Wang

**Affiliations:** 1 Liver Research Center, Beijing Friendship Hospital, Capital Medical University, National Clinical Research Center of Digestive Diseases, 95 Yong-an Road, Xicheng District, Beijing 100050, China; dacheng1717@aliyun.com (C.Z.); xxbin125@163.com (G.Z.); mahongmd@aliyun.com (H.M.); 2 Beijing Engineering Research Center for BioNanotechnology and CAS Key Laboratory for Biological Effects of Nanomaterials and Nanosafety, National Center for NanoScience and Technology, Beijing 100190, China; cyp89@126.com; 3 Beijing Entry_Exit Inspection and Quarantine Bureau, 3 Yi, Tianzhu donglu, Tianzhu Airport Industry Area, Shunyi District, Beijing 101312, China; liangxm@bjciq.gov.cn; 4 Wuhan Wawasye Nanotech Company, No. 36 Xinjiangongmen Road, Beijing 100191, China; nieleng@hotmail.com

**Keywords:** quantum dot, HBV DNA, sequencing, mutation

## Abstract

Quantum dots (QDs) are semiconductor nanoparticles with a diameter of less than 10 nm, which have been widely used as fluorescent probes in biochemical analysis and vivo imaging because of their excellent optical properties. Sensitive and convenient detection of hepatitis B virus (HBV) gene mutations is important in clinical diagnosis. Therefore, we developed a sensitive, low-cost and convenient QDs-mediated fluorescent method for the detection of HBV gene mutations in real serum samples from chronic hepatitis B (CHB) patients who had received lamivudine or telbivudine antiviral therapy. We also evaluated the efficiency of this method for the detection of drug-resistant mutations compared with direct sequencing. In CHB, HBV DNA from the serum samples of patients with poor response or virological breakthrough can be hybridized to probes containing the M204I mutation to visualize fluorescence under fluorescence microscopy, where fluorescence intensity is related to the virus load, in our method. At present, the limits of the method used to detect HBV genetic variations by fluorescence quantum dots is 10^3^ IU/mL. These results show that QDs can be used as fluorescent probes to detect viral HBV DNA polymerase gene variation, and is a simple readout system without complex and expensive instruments, which provides an attractive platform for the detection of HBV M204I mutation.

## 1. Introduction

Approximately 78 million people are chronic hepatitis B virus surface antigen (HBsAg) carriers with serological evidence, of which about 20–30 million are chronic hepatitis B (CHB) patients in China [1,2]. With long-term use of nucleos(t)ide analogs (NAs), which have shown an effective and profound suppression of viral replication in CHB therapy, the problem of hepatitis B virus (HBV) mutations under the selection of antiviral drugs increases prominently, and increases the likelihood of poor response and drug resistance in CHB patients, resulting in treatment failure and increasing the occurrence of the end-point events [3,4,5]. Although the application of entecavir (ETV) and tenofovir dipivoxil (TDF), which are the recommended antiviral agents in first-line treatment, will reduce the incidence of drug resistance [6], lamivudine (LAM), telbivudine (LdT) and adefovir dipivoxil (ADV) are widely used in the majority of primary hospitals. In addition, the number of patients with a poor response to entecavir and tenofovir are increasing. Therefore, monitoring drug resistance is critical, as is detecting HBV gene mutations and starting rescue therapy.

Currently, HBV genotyping techniques mainly include sequence analysis, microarray, reverse hybridization, restriction fragment length polymorphism, real-time PCR (polymerase chain reaction, a technique used in molecular biology to amplify a single copy or a few copies of a segment of DNA across several orders of magnitude, generating thousands to millions of copies of a particular DNA sequence), and so forth [7,8,9,10]. The direct sequencing method is the most widely used in clinics, but it has low sensitivity and only 20% of mutant species can be detected [11,12]. A wild-type sequence is used as a primer in this method, and the differences between the amplification products and the wild-type sequence are acquired by artificial comparison. In this way mutations can be found, especially in the discovery of new sites, but also as the detection of previously described mutations is relatively complex and of lower sensitivity. Thus, this method is mainly used in large hospitals with high rates of infectious diseases. The INNO-LiPA (INNO: Innogenetics N.V., Ghent, Belgium; LiPA: an assay based on the line probe assay) method [13] shows higher sensitivity than direct sequencing, and appears to be a rapid assay for the detection of HBV genotypic resistance. However, it is not suitable for clinical promotion due to the high costs involved.

With continuous improvement in fluorescence detection technology, it plays an increasingly important role in clinical diagnosis. Fluorescence analysis assays include real-time fluorescence-based quantitative polymerase chain reaction (PCR), fluorescent in situ hybridization, fluorescent images and enzyme-linked immunosorbent assay (ELISA). Real-time PCR technology has proven its usefulness in different research areas, especially in the quantification and genotyping of pathogens [14,15,16]. Fluorescence in situ hybridization (FISH) [17] is one of these rapid methods for the easy identification of microbial pathogens. As a microscopic technique, FISH has the unique potential to provide information about spatial resolution, morphology and identify key pathogens in mixed species samples. In recent years, with probe types increasing in FISH application, FISH technology not only plays a valuable role in cytogenetics, but is also widely used in oncology, virological research such as genetic diagnosis, gene mapping, and viral gene screening. ELISA has also been widely used in residue detection with high sensitivity and specificity through the specific interaction between an antibody and its corresponding antigen [18].

In addition, among the rapidly developing nanotechnology field, nano-materials have been widely used in the field of bio-analysis due to their unique optical/magnetic/electrical properties [19,20,21,22,23]. Among these nano-materials, quantum dots (QDs) have excellent optical properties such as high luminous brightness and strong light stability, which makes them suitable for use as fluorescent probes in the fields of biological image and biological detection [24,25,26,27,28]. In our study, we developed a QDs-mediated fluorescence method for detecting the hepatitis B virus gene mutation with features including low cost, high throughput, high sensitivity and accuracy.

## 2. Materials and Methods

### 2.1. Sample Preparation

Blood samples were collected from 28 CHB patients treated with LAM at the Beijing Friendship Hospital from 2009–2016. Patients treated with LAM with primary non-response (defined as less than 1 log10 IU/mL decrease in HBV DNA level from baseline at three months of therapy) and poor response (defined as a decrease in HBV DNA of more than 1 log10 IU/mL, but detectable HBV DNA by real-time PCR assay at 24 weeks of treatment) were included in the study. Exclusion criteria were as follows: co-infection with other hepatitis virus (es), and patient who had poor compliance during treatment. The serum samples were detected via sequencing. Primers were designed for the mutant gene of HBV polymerase M204I, which were confirmed by the serum samples containing M204I mutation previously detected by sequencing (performed by TIANYI HUIYUAN). The upstream and downstream primer sequences were 5′-TTGGCTTTCAGTTATATGGATGAT-3′ and 5′-TAAAAAGGGACTCAAGATGCTG-3′, respectively. The capture sequence was NH_2_-5′-TTTTTTTTTTTAAAAAGGGACTCAAGATGCTGTACAGACTTGGCC-3′, which was used to hybridize the amplified HBV DNA. Additionally, the sequence of the biotin-labeled DNA was biotin-3′-TTTTTTTTTTTCAGTTATATGGATTAT.

### 2.2. Chemical Reagents and Instruments

All chemical reagents were of analytical grade or of the highest purity available. The probes and primers that we designed were synthesized by Sangon Biotech. The proteinase K was bought from Amresco. The kits for nucleic acid extraction and PCR amplification were purchased from Wuhan Wawasye Nanotech Company and Takara Bio, respectively. The streptavidin-coated 525-nm-emission QDs (525QDs, 8 µM), size 3 nm, were bought from Qcean NanoTech (Springdale, AR, USA). The other chemical reagents include glutaraldehyde (Sinopharm Chemical Reagent Beijing Co., Ltd., Beijing, China) and the amino slides were from Gencbio (Wuhan, China). The fluorescence microscopy was from Olympus Corporation (Tokyo, Japan) and the Automatic Medical PCR Analysis System was from Shanghai Hongshi Medical Technology Co., Ltd (Shanghai, China).

### 2.3. Experimental Setup for the Detection System

#### 2.3.1. HBV DNA Extraction

The amplified DNA was obtained from the following steps:

1. The 100 μL serum samples were added into a new centrifuge tube, and then mixed with 400 μL of the lysis solution for 10 min at 70 °C.
2. To this, 300 μL of the binding solution and 20 μL of the magnetic nanobeads were added into the centrifuge tube in turn, and the mixture was left to stand for 5 min.
3. This was then washed with washing buffer I/II/III, respectively, and the supernatant was discarded after short-term centrifugation.
4. Subsequently, 30 μL of the elution was added to the centrifuge tube and mixed for 10 min at 70 °C. The DNA adsorbed on the surface of magnetic nanobeads was eluted, and the supernatant was transferred to another centrifuge tube with RNase-free for the PCR amplification.
5. The amplification was achieved for 35 cycles at 95 °C for 5 min, 95 °C for 5 s, 57 °C for 1 min, and 72 °C for 1 s.

#### 2.3.2. QDs-Mediated Fluorescent Method for the Detection of HBV M204I Mutation

To start, the amplified HBV DNA, which contains M204I mutation with viral load of 10^6^ IU/mL (the recognized international standard or copies/ml by nucleic acid testing technologies, 1 IU/mL = 5.3 copies/mL, one copy means one virus), were 10-fold serially diluted in a negative serum from 10^6^ IU/mL to 10^1^ IU/mL. The different concentrations of HBV DNA with added streptavidin-QDs were used as the positive control. The amplified HBV DNA with a viral load of 10^6^ IU/mL without streptavidin-QDs, the amplified HBV DNA without M204I mutation with a viral load of 10^6^ IU/mL with added streptavidin-QDs, and ultra-pure water were used as the negative controls, respectively.

Next, the amino slides are immersed in a 5% glutaraldehyde aqueous solution at room temperature for 2 h. The slides were washed with ultra-pure water three times and dried for 10 min at 110 °C before cooling at room temperature. The amino probe (5 μL, 20 μM) was placed on the slide, and was left at room temperature for 20 min and then stored at 4 °C overnight. 2.5μL blocking buffer including deionized formamide, SSC (SSC = 0.15 M NaCl, 0.015 M sodium citrate, pH 7.0) and denatured salmon sperm DNA were added on the glass slide and used to block the redundant site. Then, the slide was left for 30 min at room temperature.

The reactions were carried out by mixing 5 μL of amplified DNA, 5 μL of biotin labeled DNA (10 μM) at 95 °C for 10 min after being centrifuged for a short time, and immediately placed on ice for 3–5 min. Next, 5 μL of the above solution was dropped on the slide and the hybridization of the mixtures was performed on the slide by heating the reaction solution at 50 °C for 30 min, followed by adding 5 μL of water every 5–8 min. Then, 5 μL of the solution from the mixture consisting of streptavidin-QDs (10 μL) and ultrapure water (90 μL) was prepared for ligation reaction at room temperature for 30 min. After the reaction, the slide was rinsed slightly with ultra-pure water.

Finally, under the fluorescence microscope with automatic exposure mode to eliminate the interference of the slide background on the experimental results, the fluorescence signal emitted from the QDs was detected in the green channel with an excitation wavelength of 460–550 nm, focused by a 40× objective lens.

## 3. Results and Discussion

### 3.1. Mechanism of QDs-Based Fluorescent Method for HBV DNA Detection

**Figure 1:**
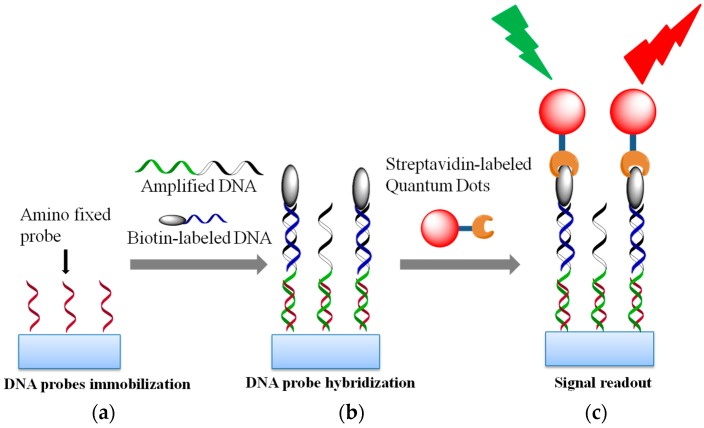
The mechanism of QDs-based fluorescent method for detection of HBV DNA. (**a**) The amino fixed probe was immobilized on a slide; (**b**) The added amplified HBV DNA can hybridize to the amino fixed probe and the biotin-labeled DNA is used to hybridize to the other side of the amplified HBV DNA; and (**c**) The streptavidin-labeled quantum dots combine with the HBV DNA of the patient to form quantum dot-DNA complexes through the reaction of biotin and streptavidin.

**Figure 2:**
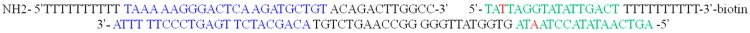
The complementarity of amplified DNA, capture and reporter probes. The mutation site was marked in red color. Amplified DNA: 3′-ATTTTTCCCTGAGTTCTACGACATGTCTGAACCGGGGGTTATGGTGATAATCCATATAACTGA-5′. Capture probe: NH_2_-5′TTTTTTTTTTTAAAAAGGGACTCAAGATGCTGTACAGACTTGGCC-3′. Reporter probe: 5′-TATTAGGTATATTGACTTTTTTTTTTT-3′-biotin.

The QDs were used as probes in this detection system. They formed a QD-DNA complex through DNA hybridization and a streptavidin-biotin system. This QD-DNA detection system was composed of streptavidin-labeled QDs, biotin-labeled DNA, and a capture. The amino-fixed probe was immobilized on an amino-modified glass-slide (Figure 1a), and the biotin-labeled DNA was used to hybridize the amplified HBV DNA. Next, the amino-fixed probe could detect the target DNA (including the M204I mutation), which had been recognized by the biotin-labeled DNA (Figure 1b). They hybridized, as shown in Figure 2. Finally, the streptavidin-labeled QDs were added to capture the complexes from the binding of biotin to streptavidin (Figure 1c). The signal from the QD-DNA was obtained from the green channel with an excitation wavelength range of 460–550 nm under fluorescence microscopy.

In this system, the accuracy of the capture sequence is the main effective factor for achieving a desirable detection limit, and hybridization time is another important factor that was optimized in this work. In addition, keeping slide clean also plays an important role, which may affects the observation results of fluorescence as the background.

### 3.2. Sensitivity of QDs-Based Fluorescent Method for HBV DNA Detection

**Figure 3:**
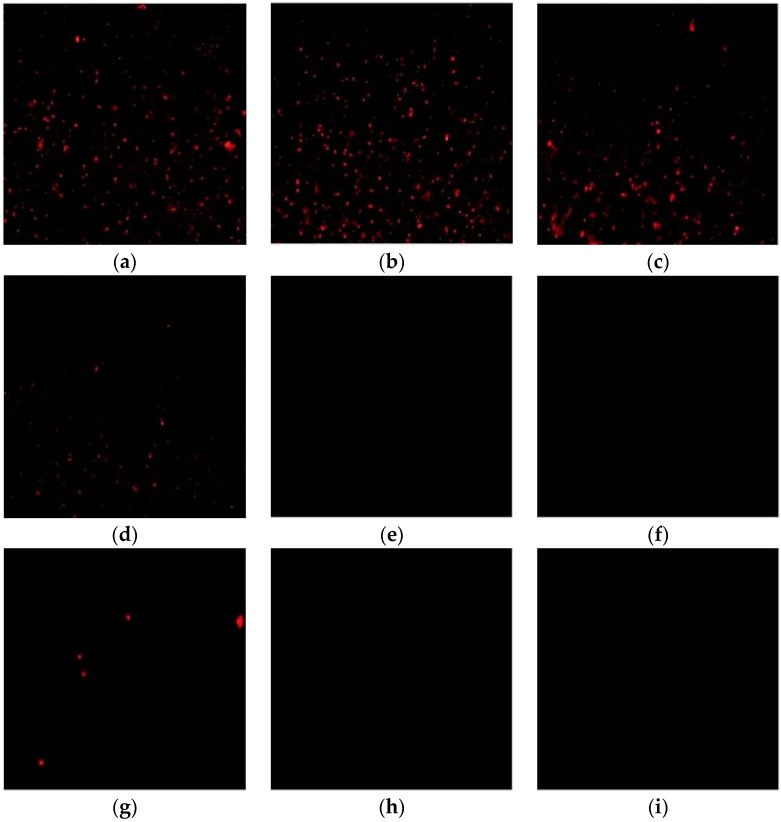
The result of QDs-based fluorescent method for detection of HBV DNA. (**a**) HBV DNA is 10^6^ IU/mL with M204I mutation; (**b**) HBV DNA is 10^5^ IU/mL with M204I mutation; (**c**) HBV DNA is 10^4^ IU/mL with M204I mutation; (**d**) HBV DNA is 10^3^ IU/mL with M204I mutation; (**e**) HBV DNA is 10^2^ IU/mL with M204I mutation; (**f**) HBV DNA is 10^1^ IU/mL with M204I mutation; (**g**) HBV DNA is 10^6^ IU/mL without M204I mutation; (**h**) HBV DNA is 10^6^ IU/m with M204I mutation, but without streptavidin-QDs; and (**i**) ultra-pure water.

The target HBV DNA were extracted and amplified from the serum of a lamivudine-resistant patient, and was detected from this system and confirmed to contain M204I mutation through direct sequencing. Figure 3 shows the representative fluorescence signals for HBV M204I mutation detection on the glass-slide with a green light under fluorescence microscopy. As seen in Figure 3, the primer can amplify the HBV DNA product containing the M204I mutation, indicating that the probe and the primer design were correct. The fluorescent light density is dependent on the concentration of the viral load; the higher the viral load, the more intensive the light spots.

Fluorescence was not observed when the amplified HBV DNA was 10^2^ IU/mL or 10^1^ IU/mL. When the viral load was less than 10^3^ IU/mL, there was no significant signal difference between the samples and the negative control, thus the limit of detection (LOD) of this method for the detection of HBV genetic variation was 10^3^ IU/mL. Furthermore, for the negative control where HBV DNA was 10^6^ IU/mL containing the M204I mutation, but without streptavidin-QDs, the result was black as there was no fluorescence to be observed under the fluorescence microscope. This suggests that the captured fluorescence was from the sample rather than any contamination. Additionally, Figure 3a,b shows similar fluorescence levels, while little fluorescence was observed in Figure 3g. We believe that this phenomenon may be related to incomplete elution, but quantitative fluorescence was not performed, making it difficult to distinguish the fluorescence levels clearly.

It is worth noting that QDs possess excellent fluorescence and photostability, and provide a simple, rapid and accurate method for the visualization detection of the HBV DNA M204I mutation.

### 3.3. Further Verification of the Accuracy of Quantum Dot Probe Hybridization Method for Detecting HBV Drug-Resistanct Sites

**Figure 4:**
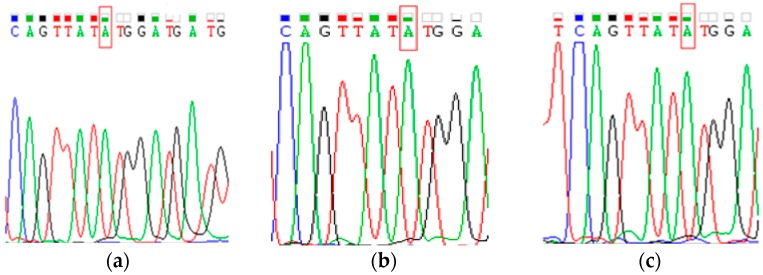
The results of DNA samples from patients with poor response were verified by direct sequencing. The DNA samples of (**a**–**c**) were extracted using the magnetic nanobeads method from the serums of patients with poor response to the nucleoside(s) treatment, and detected by the QDs-mediated fluorescent method. The results suggest that they contain the M204I mutation. The amplified products of the above three serum samples were also detected by direct sequencing, suggesting a G to A mutation.

The results show that HBV DNA polymerase gene mutation was detected by the newly established quantum dot-labeled DNA probe to detect gene mutations (Figure 4), and they show agreement between the results of the QDs-mediated fluorescent method and those detected by the direct sequencing method in patients with poor virological response during antiviral therapy, which suggests that the accuracy of the QDs-mediated fluorescent method for detecting HBV gene mutations can satisfy the need in clinical diagnosis. It was confirmed that the primers used for amplification in this study did contain the M204I mutation, which indicated that the primer sequence was correct.

### 3.4. Real Samples Analysis

The HBV M204I mutation was detected by the new method in 28 patients with chronic hepatitis B and a poor response to the nucleoside(s) treatment. The results show that the method performs well in detecting gene mutation by QD-DNA probe in patients with poor response to nucleos(t)ide analogues when compared with direct sequencing. In real serum samples analysis, 11 samples were detected to be M204I mutation-positive samples, and 17 samples were detected to be samples by the direct sequencing method. We also used the new method to detect the above serum samples and found that the abovementioned 11 samples were again detected to be M204I mutations-positive samples, while two serum samples of the 17 mutation-negative samples were in fact detected to be M204V mutations-positive samples, which were thus inconsistent with the results of direct sequencing. In order to further verify the accuracy of the new method, we employed PCR to detect these two samples, and the results confirmed that the two samples did contain the HBV M204V mutation, suggesting that the accuracy of the serum HBV DNA extraction combined with QDs-mediated fluorescent method was higher than that of the direct sequencing method.

In our previous study, we established a sensitive method of quantitative detection of HBV DNA by magnetic nanobead adsorption that was superior to other traditional extraction methods, which verified the correlation of our results showing high consistency between the test and theoretical values with an r value of 0.986 (*p* < 0.001) and reproducibility, where the average relative deviation of the magnetic nanobead method at different times was 0.505 ± 0.659. Based on this method, we established quantum dot-labeled DNA fluorescent probes to detect HBV M204I mutations, which showed a superior sensitive performance.

## 4. Conclusions

In this study, we developed a sensitive and simple fluorescent method for detecting HBV M204I mutation. The PCR step in the assay is more crucial for selectivity (mutated vs. wild sequence), as the PCR amplification step does not produce amplicon from a wild template. A second alternative to this step is the hybridization step, which can thus form a sandwich construct on a slide.

This work suggests that QDs can be used as an effective fluorescent probe to detect HBV DNA polymerase gene variation, in addition to having a simple readout system without complex and expensive instruments. Using this new method, HBV gene mutations can be detected at an early stage and rescue therapy among CHB patients can be started immediately.

Further research is required to develop a multiple fluorescent method for multiplex detection of DNA using multicolor QD nanoprobes.

## References

[1] Liang X., Bi S., Yang W., Wang L., Cui G., Cui F., Zhang Y., Liu J., Gong X., Chen Y. (2013). Reprint of: Epidemiological serosurvey of Hepatitis B in China--declining HBV prevalence due to Hepatitis B vaccination. Vaccine.

[2] Liang X., Bi S., Yang W., Wang L., Cui G., Cui F., Zhang Y., Liu J., Gong X., Chen Y. (2009). Epidemiological serosurvey of hepatitis B in China--declining HBV prevalence due to hepatitis B vaccination. Vaccine.

[3] Xing T., Xu H., Cao L., Ye M. (2017). HBeAg Seroconversion in HBeAg-Positive Chronic Hepatitis B Patients Receiving Long-Term Nucleos(t)ide Analog Treatment: A Systematic Review and Network Meta-Analysis. PLoS ONE.

[4] Yuen M.F., Ahn S.H., Chen D.S., Chen P.J., Dusheiko G.M., Hou J.L., Maddrey W.C., Mizokami M., Seto W.K., Zoulim F. (2016). Chronic Hepatitis B Virus Infection: Disease Revisit and Management Recommendations. J. Clin. Gastroenterol..

[5] Meng T., Shi X., Gong X., Deng H., Huang Y., Shan X., Shan Y., Huang A., Long Q. (2016). Analysis of the prevalence of drug-resistant hepatitis B virus in patients with antiviral therapy failure in a Chinese tertiary referral liver centre (2010–2014). J. Glob. Antimicrob. Resist..

[6] Lok A.S., Trinh H., Carosi G., Akarca U.S., Gadano A., Habersetzer F., Sievert W., Wong D., Lovegren M., Cohen D. (2012). Efficacy of entecavir with or without tenofovir disoproxil fumarate for nucleos(t)ide-naïve patients with chronic hepatitis B. Gastroenterology.

[7] Degertekin B., Hussain M., Tan J., Oberhelman K., Lok A.S. (2009). Sensitivity and accuracy of an updated line probe assay (HBV DR v.3) in detecting mutations associated with hepatitis B antiviral resistance. J. Hepatol..

[8] Sertoz R.Y., Erensoy S., Pas S., Ozacar T., Niesters H. (2008). Restriction fragment length polymorphism analysis and direct sequencing for determination of HBV genotypes in a Turkish population. New Microbiol..

[9] Valsamakis A. (2007). Molecular testing in the diagnosis and management of chronic hepatitis B. Clin. Microbiol. Rev..

[10] Shaw T., Bartholomeusz A., Locarnini S. (2006). HBV drug resistance: Mechanisms, detection and interpretation. J. Hepatol..

[11] Lok A.S., Zoulim F., Locarnini S., Bartholomeusz A., Ghany M.G., Pawlotsky J.M., Liaw Y.F., Mizokami M., Kuiken C. (2007). Hepatitis B Virus Drug Resistance Working Group. Antiviral drug-resistant HBV: Standardization of nomenclature and assays and recommendations for management. Hepatology.

[12] Lupo J., Larrat S., Hilleret M.N., Germi R., Boyer V., Nicod S., Barguès G., Leroy V., Seigneurin J.M., Zarski J.P. (2009). Assessment of selective real-time PCR for quantitation of lamivudine and adefovir hepatitis B virus-resistant strains and comparison with direct sequencing and line probe assays. J. Virol. Methods.

[13] Lok A.S., Zoulim F., Locarnini S., Mangia A., Niro G., Decraemer H., Maertens G., Hulstaert F., De Vreese K., Sablon E. (2002). Monitoring drug resistance in chronic hepatitis B virus (HBV)-infected patients during lamivudine therapy: Evaluation of performance of INNO-LiPA HBV DR assay. J. Clin. Microbiol..

[14] Klemke M., Drieschner N., Belge G., Burchardt K., Junker K., Bullerdiek J. (2012). Detection of PAX8–PPARG Fusion Transcripts in Archival Thyroid Carcinoma Samples by Conventional RT-PCR. Genes Chromosomes Cancer.

[15] Bustin S.A. (2005). Real-time, fluorescence-based quantitative PCR: A snapshot of current procedures and preferences. Expert Rev. Mol. Diagn..

[16] Navarro E., Serrano-Heras G., Castaño M.J., Solera J. (2015). Real-time PCR detection chemistry. Clin. Chim. Acta.

[17] Frickmann H., Zautner A.E., Moter A., Kikhney J., Hagen R.M., Stender H., Poppert S. (2017). Fluorescence in situ hybridization (FISH) in the microbiological diagnostic routine laboratory: A review. Crit. Rev. Microbiol..

[18] Shah K., Maghsoudlou P. (2016). Enzyme-linked immunosorbent assay (ELISA): The basics. Br. J. Hosp. Med..

[19] Zhang H., Xu T., Li C.W., Yang M. (2010). A microfluidic device with microbead array for sensitive virus detection and genotyping using quantum dots as fluorescence labels. Biosens. Bioelectron..

[20] Wang X., Lou X., Wang Y., Guo Q., Fang Z., Zhong X., Mao H., Jin Q., Wu L., Zhao H. (2010). QDs-DNA nanosensor for the detection of hepatitis B virus DNA and the single-base mutants. Biosens. Bioelectron..

[21] Kim J., Biondi M.J., Feld J.J., Chan W.C. (2016). Clinical Validation of Quantum Dot Barcode Diagnostic Technology. ACS Nano.

[22] Gerion D., Parak W.J., Williams S.C., Zanchet D., Micheel C.M., Alivisatos A.P. (2002). Sorting fluorescent nanocrystals with DNA. J. Am. Chem. Soc..

[23] Huang S., Qiu H., Xiao Q., Huang C., Su W., Hu B. (2013). A Simple QD-FRET Bioprobe for Sensitive and Specific Detection of Hepatitis B Virus DNA. J. Fluoresc..

[24] Shamsipur M., Nasirian V., Mansouri K., Barati A., Veisi-Raygani A., Kashanian S. (2017). A highly sensitive quantum dots-DNA nanobiosensor based on fluorescence resonance energy transfer for rapid detection of nanomolar amounts of human papillomavirus 18. J. Pharm. Biomed. Anal..

[25] Foubert A., Beloglazova N.V., Rajkovic A., Sas B., Madder A., Goryacheva I.Y., Saeger S.D. (2016). Bioconjugation of quantum dots: Review & impact on future application. TrAC Trends Anal. Chem..

[26] Kuang H., Zhao Y., Ma W., Xu L., Wang L., Xu C. (2011). Recent developments in analytical applications of quantum dots. Trends Anal. Chem..

[27] Zhang P., Liu S., Gao D., Hu D., Gong P., Sheng Z., Deng J., Ma Y., Cai L. (2012). Click-functionalized compact quantum dots protected by multidentate-imidazole ligands: Conjugation-ready nanotags for living-virus labeling and imaging. J. Am. Chem. Soc..

[28] Algar W.R., Tavares A.J., Krull U.J. (2010). Beyond labels: A review of the application of quantum dots as integrated components of assays, bioprobes, and biosensors utilizing optical transduction. Anal. Chim. Acta.

